# α_1_-adrenoceptor stimulation ameliorates lipopolysaccharide-induced lung injury by inhibiting alveolar macrophage inflammatory responses through NF-κB and ERK1/2 pathway in ARDS

**DOI:** 10.3389/fimmu.2022.1090773

**Published:** 2023-01-06

**Authors:** Zhukai Cong, Cui Yang, Zhaojin Zeng, Changyi Wu, Feng Zhao, Ziyuan Shen, Han Xiao, Xi Zhu

**Affiliations:** ^1^ Department of Critical Care Medicine, Peking University Third Hospital, Beijing, China; ^2^ Department of Anaesthesiology, Peking University Third Hospital, Beijing, China; ^3^ Department of Cardiology and Institute of Vascular Medicine, Peking University Third Hospital, Beijing, China; ^4^ National Health Commission (NHC) Key Laboratory of Cardiovascular Molecular Biology and Regulatory Peptides, Beijing, China; ^5^ Key Laboratory of Molecular Cardiovascular Science, Ministry of Education, Beijing, China; ^6^ Key Laboratory of Cardiovascular Receptors Research, Beijing, China

**Keywords:** acute respiratory distress syndrome, α_1_ adrenergic receptor, alveolar macrophage, inflammation, NF-κb

## Abstract

**Introduction:**

Catecholamines such as norepinephrine or epinephrine have been reported to participate in the development of acute respiratory distress syndrome (ARDS) by activating adrenergic receptors (ARs). But the role of α1-AR in this process has yet to be elucidated.

**Methods:**

In this study, ARDS mouse model was induced by intratracheal instillation of lipopolysaccharide. After treatment with α1-AR agonist phenylephrine or antagonist prazosin, lung pathological injury, alveolar barrier disruption and inflammation, and haemodynamic changes were evaluated. Cytokine levels and cell viability of alveolar macrophages were measured in vitro. Nuclear factor κB (NF-κB), mitogen-activated protein kinase, and Akt signalling pathways were analysed by western blot.

**Results:**

It showed that α1-AR activation alleviated lung injuries, including reduced histopathological damage, cytokine expression, and inflammatory cell infiltration, and improved alveolar capillary barrier integrity of ARDS mice without influencing cardiovascular haemodynamics. *In vitro* experiments suggested that α1-AR stimulation inhibited secretion of TNF-α, IL-6, CXCL2/MIP-2, and promoted IL-10 secretion, but did not affect cell viability. Moreover, α1-AR stimulation inhibited NF-κB and enhanced ERK1/2 activation without significantly influencing p38, JNK, or Akt activation.

**Discussion:**

Our studies reveal that α1-AR stimulation could ameliorate lipopolysaccharide-induced lung injury by inhibiting NF-κB and promoting ERK1/2 to suppress excessive inflammatory responses of alveolar macrophages.

## Introduction

Acute respiratory distress syndrome (ARDS) is a common critical illness characterized by acute hypoxic respiratory insufficiency or failure, often requiring hospitalisation in an intensive care unit (1). The life-threatening illness can be caused by a variety of non-cardiogenic factors, including pneumonia, sepsis, and trauma (2). Because of its multifactorial aetiology and complex pathogenesis, ARDS shows great heterogeneity across different subpopulations of patients (3). Although prior studies have made considerable progress in understanding the pathogenesis of ARDS, no effective drug interventions are currently available (4) and the morbidity and mortality rates remain high (5, 6) . This is especially important with the outbreak of Corona Virus Disease 2019 (COVID-19), the severe stage of which can lead to ARDS, bringing tremendous challenges to clinical treatment and basic research (7). Therefore, it is urgent to further explore the pathogenesis of ARDS and identify feasible therapeutic strategy.

An uncontrolled inflammatory response is generally regarded as the core mechanism resulting in diffuse alveolar damage and lung oedema (8). Clinical evidence suggests that cytokines and inflammatory cells in plasma or bronchoalveolar lavage fluid (BALF) of patients with ARDS are usually increased, and associated with mortality (9). Accordingly, regulation of the immune inflammatory response is considered a potential treatment strategy for ARDS (8).

In addition to its established role as a regulator of the cardiovascular system, a growing body of evidence indicates that sympathetic nervous system (SNS) is an integrative interface between the nervous system and the immune system (10). SNS dysfunction is common during sepsis (11, 12) and sepsis-induced complication, like ARDS (13), and can influence disease progression (14). Norepinephrine (NE), an important neurotransmitter released from the SNS, is an essential vasoactive agent used clinically to treat septic shock (15). Recent evidence suggests that NE could regulates inflammatory response of immune cells and participate in the development of ARDS. And our previous studies showed that NE could inhibit activation of alveolar macrophages and alleviate lung inflammation in ARDS mice induced by lipopolysaccharide (LPS) (16), however, the underlying mechanism is unclear. Adrenergic receptors (ARs) including α_1_-AR, α_2_-AR, and β-AR mediate the effects of NE (17). Previous studies showed that blockade of α_2_-AR (16, 18, 19) or stimulation of β-AR (20–22) could alleviate lung injury by reducing inflammation. However, the role and mechanism of α_1_-AR in ARDS is still not fully understood. *In vivo* experiments indicate that phenylephrine (PE), a specific agonist of α_1_-AR, is favourable for protecting the structural and functional integrity of alveolar-capillary barriers (23, 24). Other *in vitro* studies suggest that α_1_-AR stimulation could influence cytokines expression of inflammatory cells (25, 26). Therefore, we explored the effect of α_1_-AR activation on lung inflammation in a mouse model of ARDS. We hypothesized that the beneficial effect of α_1_-AR for ARDS arise from its ability to alleviate inflammation through effects on alveolar macrophages.

## Materials and methods

### Animals

Male C57BL/6J mice (8–12 weeks old) were purchased from and maintained in the Department of Laboratory Animal Science at Peking University Health Science Centre (Peking, China). Mice were kept on a 12-h light/dark cycle with *ad libitum* access to standard diet and water. All animal experimental procedures were approved by the Animal Care and Scientific Committee of Peking University Health Science Centre (Approval No: SA2020336).

### Animal model and experimental protocol

**Figure 1:**
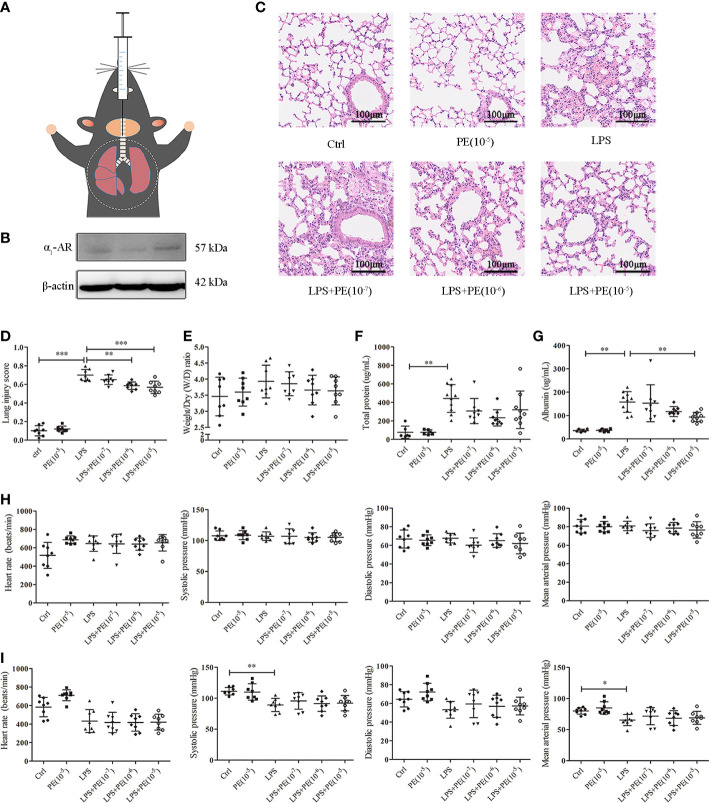
Phenylephrine (PE) attenuated lung pathological injury and alveolar capillary barrier disruption without influencing the cardiovascular haemodynamics of ARDS mice. Mice were given an intratracheal instillation of 2 mg/kg lipopolysaccharide (LPS) to induce ARDS, and PE was injected into trachea 20 min before LPS stimulation. Lung tissues and bronchoalveolar lavage fluid (BALF) were collected 24 h after LPS stimulation. **(A)** Method for intratracheal instillation of LPS or PE in mice. **(B)** Expression of α_1_-AR in lung tissues of mice. **(C)** Haematoxylin and eosin staining of lung slices. Scale bar = 100 μM. **(D)** Histology scores of lungs were judged according to guidelines of the American Thoracic Society. **(E)** Wet/dry weight ratio of lung tissues. **(F)** Levels of total protein in BALF. **(G)** Levels of albumin in BALF. **(H)** Heart rate (HR), systolic blood pressure (SP), diastolic blood pressure (DP), and mean artery pressure (MAP) of mice were monitored before establishing ARDS model. **(I)** HR, SP, DP, and MAP of mice were monitored 24 h after establishing ARDS model. Data are represented as the mean ± SD, n = 6–10 per group. *p < 0.05, **p < 0.01, ***p < 0.001.

Mice were anaesthetized by intraperitoneal administration of 1% pentobarbital sodium (70 mg/kg, Sigma-Aldrich, St. Louis, MO, USA). As shown in **Figure 1A**, after anaesthesia, the ARDS mouse model was established by intratracheal instillation of 2 mg/kg LPS (*Escherichia coli* 0111:B4, Sigma-Aldrich) in 50 µL of phosphate-buffered saline (PBS). To examine the effect of α_1_-AR on lung injury of ARDS mice, animal experiments were divided into two parts. In the first part, 20 min before LPS stimulation, varying concentrations of the α_1_-AR agonist PE (10^-7^–10^-5^ M, Selleck Chemicals, Houston, TX, USA) in 50 µL of PBS were injected into the tracheae of ARDS mice. In the second part, 20 min before PE (10^-5^ M) intervention, the α_1_-AR antagonist prazosin (PRA, 10^-5^ M) in 50 µL of PBS was injected into the tracheae of ARDS mice. Simultaneously, control groups were treated with 50 µL of PBS. Mice were sacrificed 24 h after LPS stimulation, and lung tissues and BALF were obtained.

### Haemodynamic parameter monitoring

Mice were placed in a noninvasive blood pressure monitor (Softron Biotechnology, Beijing, China) for 10 min daily to become accustomed to the environment for 3 days before establishing the ARDS model. Heart rate (HR), systolic blood pressure (SP), diastolic blood pressure (DP), and mean artery pressure (MAP) of mice were monitored before and 24 h after establishing ARDS model to estimate haemodynamic changes.

### BALF preparation and cell counts

Bronchoalveolar lavage was carried out with 1 mL of ice-cold PBS three times. The collected BALF was centrifuged at 1000 × g at 4°C for 5 min. BALF supernatant was stored at -80°C for subsequent detection. One part of the cell pellet was used for total cell counts with a haemocytometer, while the other part was used for differential counts of inflammatory cells by Wright-Giemsa staining (Nanjing Jiancheng Bioengineering Institute, Nanjing, China).

### Histology

The inferior lobe of the right lung from mice without bronchoalveolar lavage was fixed in 4% paraformaldehyde for 24–48 h, dehydrated in an ascending gradient of alcohol, embedded in paraffin, and sliced into 5-µm sections. After staining with haematoxylin and eosin, lung tissues were scanned by a digital pathology microscope (Hamamatsu Photonics, Hamamatsu City, Japan). Histological changes were assessed by a blinded investigator according to a standardized histology scoring system published by American Thoracic Society (27).

### Determination of lung wet/dry weight ratio

The left lung lobe from mice without bronchoalveolar lavage was weighed to record the wet weight. Next, the lung lobe was dried in an oven at 65°C for 48 h until all moisture was removed, and the dry weight was measured. The wet/dry weight ratio was calculated as a measure of the severity of pulmonary oedema.

### Cell culture and treatment

The murine alveolar macrophage cell line MH-S was purchased from Bio-Rad Laboratories (Hercules, CA, USA). Cells were cultured in RPMI-1640 medium (Biological Industries, Kibbutz Beit Haemek, Israel) with 10% foetal bovine serum (Biological Industries) at 37°C in the presence of 5% CO_2_.

For LPS activation, MH-S cells were stimulated with 100 ng/mL LPS (Sigma-Aldrich). In accordance with the animal experiment design, during the first part, MH-S cells were incubated with PE (10^-8^–10^-5^ M) for 30 min and then stimulated with LPS for 6 h. In the second part, cells were pre-incubated with PRA (10^-5^ M) for 30 min, followed by PE (10^-5^ M) for 30 min, and then stimulated with LPS for 6 h. Cell supernatants and pellets were collected for evaluation.

### Cytokine and albumin assays

Inflammatory cytokines tumour necrosis factor α (TNF-α), interleukin (IL)-6, IL-10, and chemokine (C-X-C motif) ligand 2/macrophage inflammatory protein 2 (CXCL2/MIP-2) in BALF and cell supernatant were detected by enzyme-linked immunosorbent assay (ELISA) duoset kits (R&D Systems, Minneapolis, MN, USA) according to the kit manufacturer’s instructions. The concentration of albumin in BALF was also measured by ELISA (Elabscience, Wuhan, China).

### Cell viability assay

Cell Counting Kit-8 (CCK-8) and calcein-AM/PI double staining assays (Yeasen Biotechnology, Shanghai, China) were used to measure cell viability. For CCK-8, MH-S cells were seeded into 96-well plates at a density of 5 × 10^3^ cells/well. After treatment with PE, PRA, or LPS, cells were incubated with 10 μL of CCK-8 reagent for 2 h, and the absorbance value of each well was measured using an Automatic Microplate Reader (Thermo Fisher Scientific, Waltham, MA, USA) at 450 nm. For calcein-AM/PI double staining, MH-S cells were seeded into 48-well plates at a density of 2 × 10^5^ cells/well. After identical administration of PE, PRA, or LPS, a mixture of calcein-AM and PI were added into each well for 15 min. Finally, live cells (yellow-green fluorescence) and dead cells (red fluorescence) were simultaneously observed at a 490 ± 10 nm excitation wavelength under a fluorescence microscope (Leica, Wetzlar, Germany).

### Immunofluorescence staining

MH-S cells were seeded into 15-mm glass bottom dishes (Nest Biotechnology, Jiangsu, China) at a density of 2 × 10^5^ cells. After fixation with 4% paraformaldehyde, cells were permeabilized with 0.5% Triton X-100, blocked in blocking buffer, and incubated overnight with an anti-α_1_-AR antibody (1:100; Abcam, Cambridge, UK) at 4°C. The following day, cells were incubated with a fluorochrome-conjugated secondary antibody [1:500; Cell Signaling Technology (CST), Danvers, MA, USA] for 1 h at room temperature protected from light. DAPI reagent (Solarbio, Beijing, China) was used for nuclei staining. Finally, specimens were observed under a fluorescence microscope (Leica).

### Western blot analysis

Lung tissues were first ground into a single-cell suspension. Obtained lung cells and MH-S cells were lysed in RIPA buffer (Applygen, Beijing, China) for protein extraction. After determining protein concentrations with a bicinchoninic acid colorimetric assay kit (Applygen), protein samples were separated by 10% sodium dodecyl sulfate-polyacrylamide gel electrophoresis, and then transferred to polyvinylidene fluoride membranes (MilliporeSigma, Burlington, MA, USA). Membranes were blocked in 1× Tris-buffered saline containing Tween with 5% w/v non-fat dry milk for 1 h at room temperature and incubated overnight at 4°C with primary antibodies against p-p65 (1:1,000; CST), p65 (1:1,000; CST), p-p38 (1:1,000; CST), p38 (1:1,000; CST), p-ERK1/2 (1:1,000; CST), ERK1/2 (1:1,000; CST), p-JNK (1:1,000; CST), JNK (1:1,000; CST), p-Akt (1:1,000; CST), Atk (1:1,000; CST), α_1_-AR (1:1,000; Abcam), and β-actin (1:5,000; Applygen). After three washes, membranes were incubated with secondary antibodies for 1 h at room temperature. Protein bands were visualized using a chemiluminescence image analysis system (Tanon Science and Technology, Shanghai, China).

### Statistical analysis

All experiments were repeated at least five times, and statistical analysis was conducted using SPSS 22.0 software (IBM SPSS, Chicago, IL, USA). Data conforming to a normal distribution are presented as mean ± standard deviation (SD), and were analysed with one-way analysis of variance (ANOVA) followed by Bonferroni *post hoc* test or Welch’s ANOVA followed by Dunnett’s T3 *post hoc* test. Experimental data not conforming to a normal distribution are presented as median (25%, 75%), and were analysed by non-parametric test. *P* values < 0.05 were considered statistically significant.

## Results

### α_1_-AR agonist PE attenuated lung injury of ARDS mice

Histopathological lesions provide intuitive and reliable results (28). To evaluate the effect of α_1_-AR on ARDS, expression of α_1_-AR was first confirmed in lung tissues of mice without intervention (**Figure 1B**). Evaluation of histopathological lesions provide intuitive and reliable results (28). Histology showed that intratracheal instillation of LPS induced evident lung injury, including alveolar septum thickening, fusion of alveoli, interstitial oedema, and inflammatory cell infiltration (**Figure 1C**). However, treatment with the α_1_-AR specific agonist PE mitigated these changes. Compared with the LPS group, PE at both of the concentration of 10^-6^ and 10^-5^ M treatment significantly improved the degree of lung injury (**Figure 1D**), as assessed by the standardized histology scoring system (27). Alveolar capillary barrier disruption, another key indicator of ARDS, is usually evaluated by measuring the wet/dry weight ratio of lung tissues, as well as total protein and/or albumin concentration in BALF. Our results show that LPS stimulation produced no evident changes in the wet/dry weight ratio of lung tissues (**Figure 1E**), however it increased total protein and albumin concentration in BALF, suggesting the occurrence of lung injury (**Figures 1E, G**). Although PE had no significant effect on total protein concentrations in BALF, albumin concentrations (an indicator with higher sensitivity) were evidently reduced following intervention with PE (10^- 5^ M).

### PE did not influence the cardiovascular haemodynamics of ARDS mice

Clinically, PE serves as a vasoactive agent typically used to increase patient blood pressure (29). Therefore, cardiovascular haemodynamic parameters were monitored in this study. As shown in **Figure 1H**, we first observed HR, SP, DP, and MAP of mice before establishing the ARDS model in mice. As expected, no changes were observed in the group without treatment, indicating that our monitoring method was stable and reliable. After stimulation with LPS, SP and MAP were significantly reduced compared with those in the control group, although there were no differences in HR or DP. PE treatment did not influence any of these indicators (**Figure 1I**).

### PE ameliorated lung inflammation in ARDS mice

**Figure 2:**
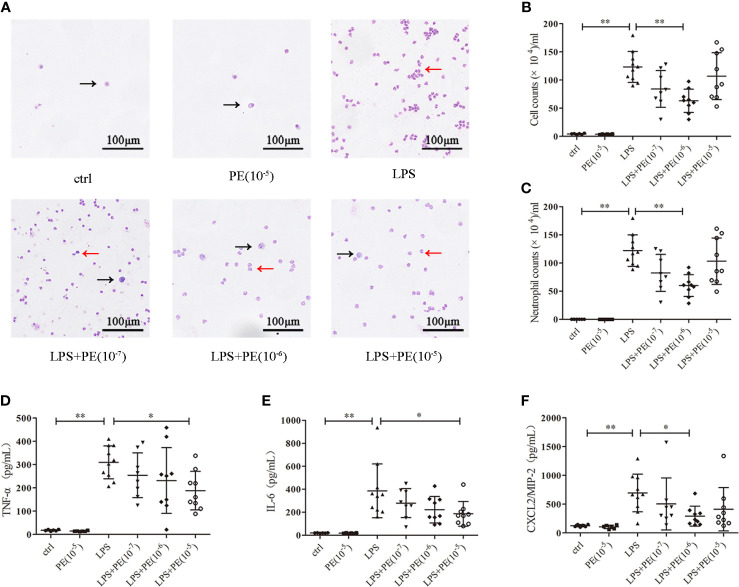
Phenylephrine (PE) ameliorated lung inflammation in ARDS mice. Mice were given an intratracheal instillation of 2 mg/kg lipopolysaccharide (LPS) to induce ARDS, and PE was injected into trachea 20 min before LPS stimulation. Lung tissues and bronchoalveolar lavage fluid (BALF) were collected 24 h after stimulation with LPS. **(A)** Inflammatory cells in BALF stained by Wright-Giemsa stain. Scale bar = 100 μM. **(B)** Counts of total inflammatory cells in BALF. **(C)** Counts of neutrophils in BALF. Levels of TNF-α **(D)**, IL-6 **(E)**, and CXCL2/MIP-2 **(F)** in BALF. Data are represented as the mean ± SD, n = 6–10 per group. *p < 0.05, **p < 0.01.

Excessive inflammatory responses are generally considered the major underlying pathogenesis of ARDS (2). Therefore, we detected the number of exudated inflammatory cells and cytokine levels in BALF. As shown in **Figure 2A**, LPS induced infiltration of large numbers of inflammatory cells (**Figure 2B**), especially neutrophils (**Figure 2C**), into lung tissues. Treatment with PE (10^-6^ M) obviously decreased the number of total cells and neutrophils in BALF. Similarly, inflammatory cytokines TNF-α (**Figure 2D**), IL-6 (**Figure 2E**), and CXCL-2/MIP-2 (**Figure 2F**) in BALF were dramatically increased after LPS stimulation, but inhibited by treatment with PE.

### PE inhibited inflammatory responses of alveolar macrophages without influencing cell viability

**Figure 3:**
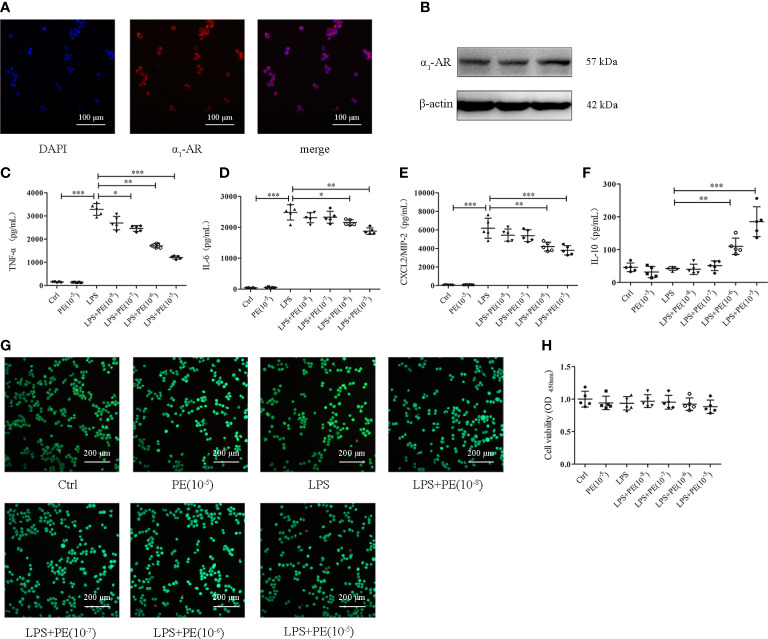
Phenylephrine (PE) inhibited inflammation in lipopolysaccharide (LPS)-activated murine alveolar macrophages without influencing cell viability. MH-S cells were incubated with PE (10^-8^-10^-5^ M) for 30 min, followed by LPS (100 ng/mL) for 6 h Expression of α_1_-AR in MH-S cells was detected by immunofluorescence **(A)** and western blotting **(B)**. Levels of TNF-α **(C)**, IL-6 **(D)**, CXCL2/MIP-2 **(E)**, and IL-10 **(F)** released from MH-S cells. **(G)** Live and dead cells were stained by calcein-AM and propidium iodide, respectively. Scale bar = 200 μM. **(H)** Cell viability was measured by CCK-8 assay. Data are represented as the mean ± SD, n = 5 per group. *p < 0.05, **p < 0.01, ***p < 0.001.

Alveolar macrophages are a primary cell type in the lung inflammatory processes (30). Interestingly, we detected α_1_-AR expression in alveolar macrophages without intervention (**Figures 3A, B**). To determine whether the effect of PE on lung inflammation involved the effects on alveolar macrophages, we examined cytokine levels in the murine alveolar macrophage line MH-S following LPS stimulation and intervention with PE. The results show that PE inhibited the secretion of TNF-α (**Figure 3C**) at the concentration of 10^-7^, 10^-6^ and 10^-5^ M, while concentrations of 10^-6^ and 10^-5^ M PE inhibited the secretion of IL-6 (**Figure 3D**) and CXCL2/MIP-2 (**Figure 3E**), and significantly promoted the secretion of IL-10 (**Figure 3F**). In addition, we observed the effect of PE on cell viability by calcein-AM/PI double staining and CCK-8 assay. The results show that there were no significant differences among the groups (**Figures 3G, H**), suggesting that the anti-inflammatory effect of PE did not involve effects on cell viability.

### PE inhibited NF-κB activation and promoted activation of ERK1/2 in LPS-stimulated alveolar macrophages

**Figure 4:**
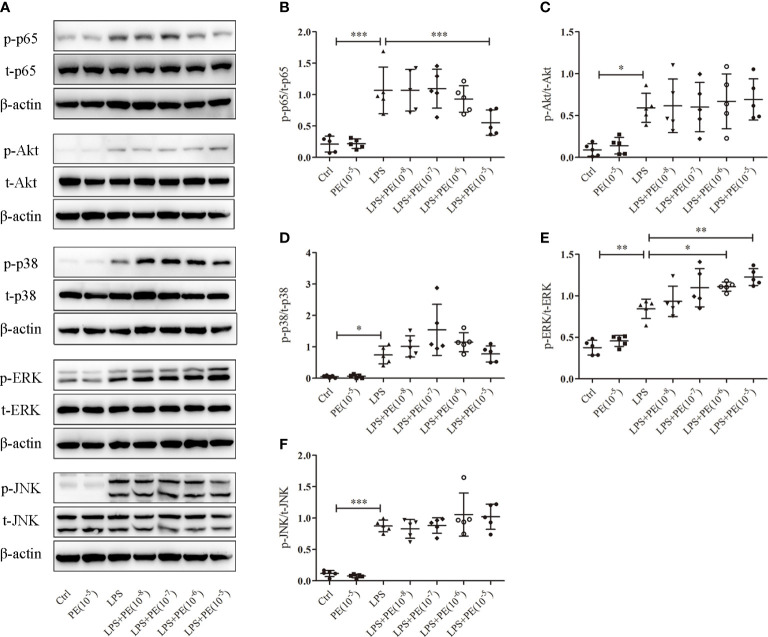
Phenylephrine (PE) suppressed NF-κB activation and enhanced ERK1/2 activation in alveolar macrophages stimulated by lipopolysaccharide (LPS). MH-S cells were incubated with PE (10^-8^-10^-5^ M) for 30 min, followed by LPS (100 ng/mL) for 30 min. **(A)** Western blotting was used to evaluate activation of p65, Akt, p38, ERK1/2, and JNK. Phosphorylation of p65 **(B)**, Akt **(C)**, p38 **(D)**, ERK1/2 **(E)**, and JNK **(F)** were analysed according to grey values. Data are represented as the mean ± SD, n = 5 per group. *p < 0.05, **p < 0.01, ***p < 0.001.

NF-κB, Akt, and MAPK signalling pathways are important for regulating inflammatory responses (31). Increased phosphorylation levels of p65 and Akt represent activation of NF-κB and Akt signaling pathways respectively (31). The MAPKs in mammals include p38, ERK and JNK which are serine-threonine protein kinases that regulate various cellular functions including inflammation (32). As shown in **Figure 4**, LPS stimulation caused the activation of NF-κB, Akt, and p38, ERK1/2 and JNK. However, PE (10^-5^M) treatment could inhibit NF-κB activation (**Figure 4B**) and further promote ERK1/2 activation (**Figure 4E**), but had no effect on activation of Akt (**Figure 4C**), p38 (**Figure 4D**), or JNK (**Figure 4F**) by LPS.

### α_1_-AR specific blocker PRA reversed the effect of PE on lung injury in ARDS mice

**Figure 5:**
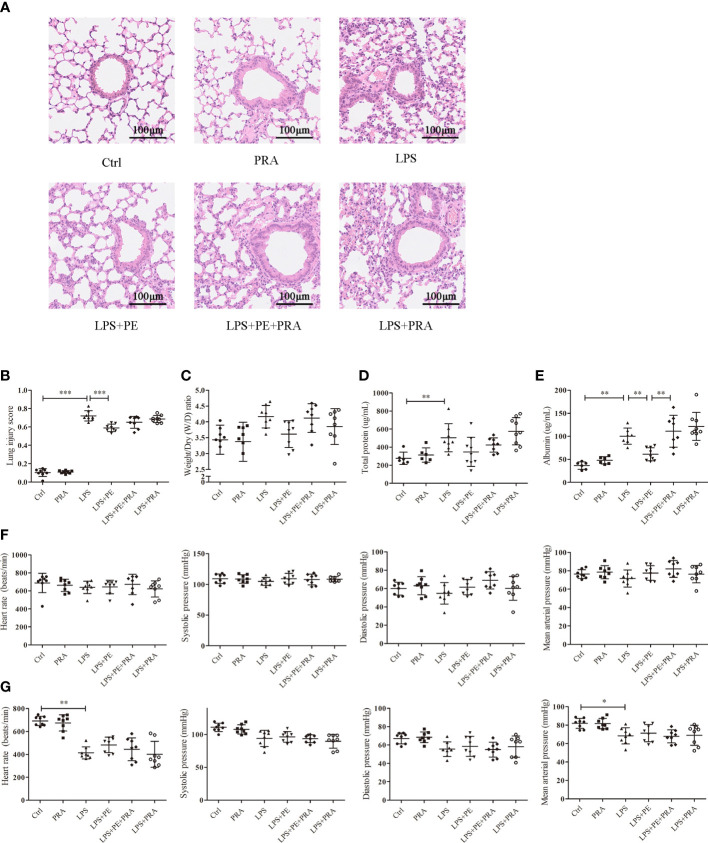
Prazosin (PRA) reversed the effect of phenylephrine (PE) on lung pathological injury and alveolar capillary barrier disruption without influencing the cardiovascular haemodynamic of ARDS mice. Mice were given an intratracheal instillation of 2 mg/kg lipopolysaccharide (LPS) to induce ARDS. PRA and PE were also injected into the trachea 40 min or 20 min before LPS stimulation. Lung tissues and bronchoalveolar lavage fluid (BALF) were collected 24 h after LPS stimulation. **(A)** Haematoxylin and eosin staining of lung slices. Scale bar = 100 μM. **(B)** Histology scores of lungs were judged according to guidelines of the American Thoracic Society. **(C)** Wet/dry weight ratio of lung tissues. **(D)** Levels of total protein in BALF. **(E)** Levels of albumin in BALF. **(F)** Heart rate (HR), systolic blood pressure (SP), diastolic blood pressure (DP), and me an artery pressure (MAP) of mice were monitored before establishing ARDS model. **(G)** HR, SP, DP, and MAP of mice were monitored 24 h after establishing ARDS model. Data are represented as the mean ± SD, n = 6–9 per group. *p < 0.05, **p < 0.01, ***p < 0.001.

To further determine whether the protective effect of PE on lung injury of ARDS mice occurred through activation of α_1_-AR, we administered the α_1_-AR specific blocker PRA to ARDS mice along with PE intervention. A concentration of 10^-5^ M PE was chosen for these animal experiments, because it could more thoroughly alleviate lung injury according to experimental results described above. Although no significant differences were observed in lung injury scores (**Figure 5B**), W/D ratios (**Figure 5C**), or total protein levels (**Figure 5D**) between LPS+PE and LPS+PE+PRA groups, PRA aggravated alveolar septum thickening and the fusion of alveoli (**Figure 5A**), and reversed the inhibitory effect of PE on leakage of albumin into alveoli (**Figure 5E**). These results suggest that PRA reversed the effect of PE on lung injury of ARDS mice to some extent.

### PRA did not influence the cardiovascular haemodynamics of ARDS mice

PRA works as a specific blocker of α_1_-AR and is used to treat high blood pressure (33). Therefore, we monitored its effect on the cardiovascular haemodynamics of ARDS mice. Consistent with previous results (**Figure 1H, I**), HR, SP, DP, and MAP of mice before establishing ARDS model were unchanged without any treatment (**Figure 5F**). After stimulation with LPS, HR and MAP were significantly reduced compared with those in the control group, although there were no differences in SP or DP. Indeed, neither PE nor PRA influenced these indicators (**Figure 5G**).

### PRA reversed the effect of PE on lung inflammation in ARDS mice

**Figure 6:**
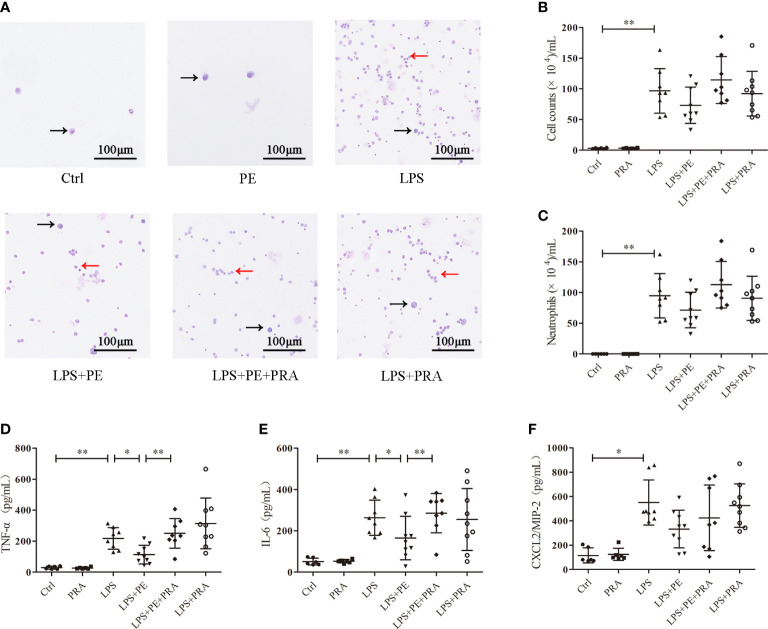
Prazosin (PRA) reversed the effect of phenylephrine (PE) on lung inflammation in ARDS mice. Mice were given an intratracheal instillation of 2 mg/kg lipopolysaccharide (LPS) to induce ARDS. PRA (10^-5^ M) and PE (10^-5^ M) were also injected into the trachea 40 min or 20 min before LPS stimulation. Lung tissues and bronchoalveolar lavage fluid (BALF) were collected 24 h after LPS stimulation. **(A)** Inflammatory cells in BALF stained by Wright-Giemsa stain. Scale bar = 100 μM. **(B)** Counts of total inflammatory cells in BALF. **(C)** Counts of neutrophils in BALF. Levels of TNF-α **(D)**, IL-6 **(E)**, and CXCL2/MIP-2 **(F)** in BALF. Data are represented as the mean ± SD, n = 6–10 per group. *p < 0.05, **p < 0.01.

The effect of PRA on lung inflammation was also evaluated. From the staining assay, further treatment of PRA increased numbers of inflammatory cells in the BALF of ARDS mice compared with the LPS+PE group (**Figure 6A**). Although image analysis revealed a similar trend, the difference was not statistically significant (**Figure 6B, C**). However, our results show that PRA could reverse the inhibitory effect of PE on expression of TNF-α (**Figure 6D**) and IL-6 (**Figure 6E**) in lung tissues of ARDS mice. Consistent with previous results (**Figure 2F**), neither PE (10^-6^ M) nor PRA affected expression of CXCL-2/MIP-2 (**Figure 6F**).

### PRA reversed the effect of PE on inflammatory responses of alveolar macrophages without influencing cell viability

**Figure 7:**
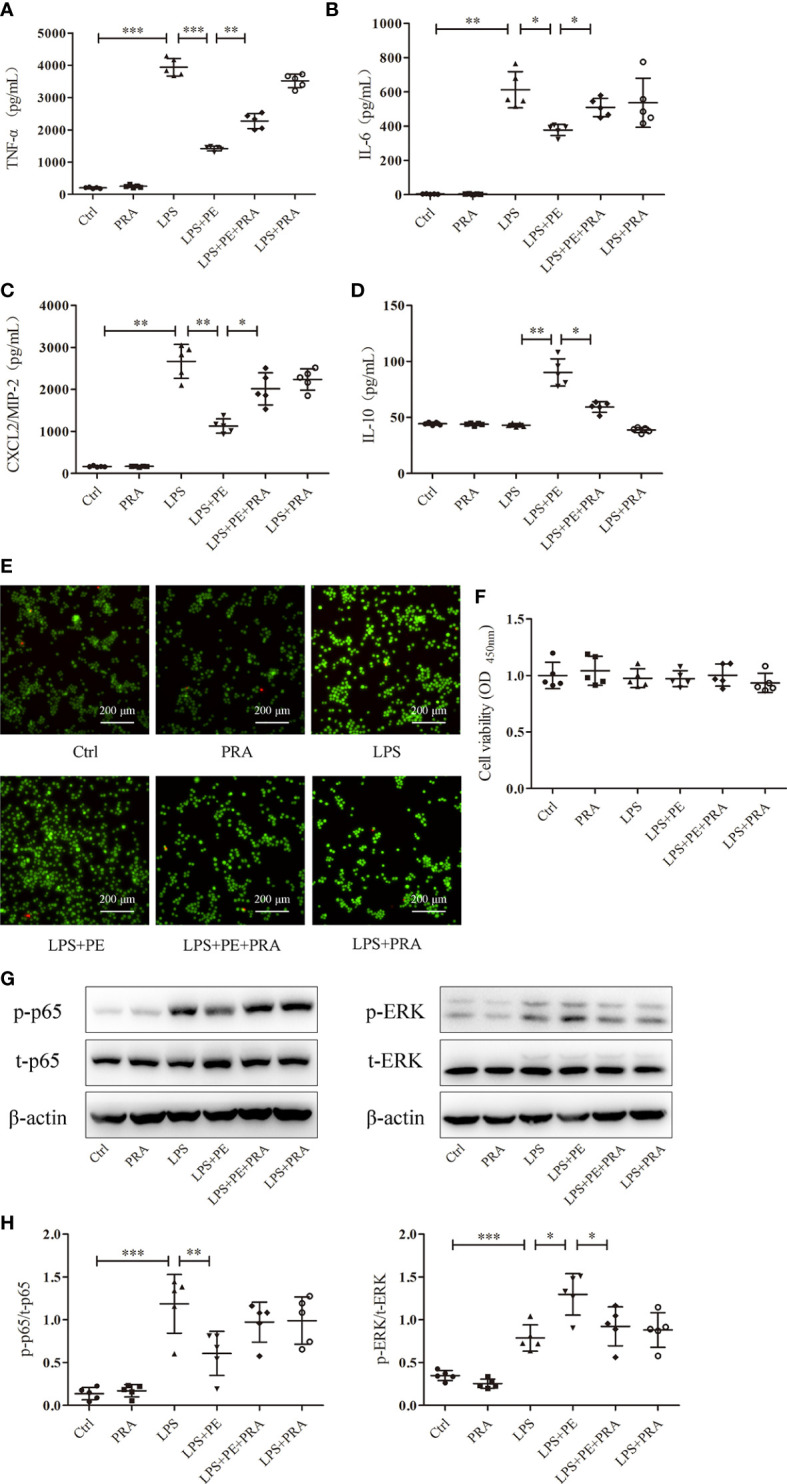
Prazosin (PRA) reversed the effect of phenylephrine (PE) on inflammation in lipopolysaccharide (LPS)-activated murine alveolar macrophages without influencing cell viability by suppressing NF-κB activation and enhancing ERK1/2 activation. MH-S cells were incubated with PRA (10^-5^ M) for 30 min, followed by PE (10^-5^ M) treatment for 30 min, and then LPS (100 ng/mL) for 6 h Levels of TNF-α **(A)**, IL-6 **(B)**, CXCL2/MIP-2 **(C)**, and IL-10 **(D)** released from MH-S cells. **(E)** Live and dead cells were stained by calcein-AM and propidium iodide, respectively. Scale bar = 200 μM. **(F)** Cell viability was measured by CCK-8 assay. **(G)** Western blotting was used to evaluate activation of p65 and ERK1/2. **(H)** Phosphorylation of p65 and ERK1/2 were analysed according to grey values. Data are represented as the mean ± SD, n = 5 per group. *p < 0.05, **p < 0.01, ***p < 0.001.

We also observed the effect of PRA on inflammatory responses of alveolar macrophages. As shown in **Figures 7A–D**, PRA partially reversed the effect of PE on secretion of TNF-α, IL-6, CXCL2/MIP-2, and IL-10 from alveolar macrophages after stimulation with LPS. Calcein-AM/PI double-staining and CCK-8 assays were used to rule out of the possibility that the effect of PRA on inflammatory responses of alveolar macrophages did not result from influences on cell viability (**Figures 7E, F**).

### PRA reversed the effects of PE on activation of NF-κB and ERK1/2 in LPS-stimulated alveolar macrophages

Although we detected many signal molecules related to inflammation, only NF-κB and ERK1/2 exhibited changes following intervention with PE. To further determine whether this phenomenon was attributed to activation of α_1_-AR by PE, the activation of NF-κB and ERK1/2 was observed following treatment with PRA. As shown in **Figures 7G, H**, PE promoted the activation of ERK1/2, while further treatment with PRA partially reversed the effect of PE. Although there was no significant statistical difference in NF-κB activation between LPS+PE and LPS+PE+PRA groups, PRA treatment still increased expression of p-p65 to some degree (according to the protein band).

## Discussion

ARDS is a life-threatening condition with high morbidity and mortality rates for patients in the intensive care unit (1). Although massive efforts have been made, current treatment is still limited to mechanical ventilation (34), fluid management (35), and other supportive therapies (36, 37); at present, there is no proven pharmacotherapy (4). To counter the problems above, preliminary exploration was carried out through animal and cell experiments in this study. The results show that α_1_-AR stimulation alleviated lung injury of ARDS mice, including inhibition of lung inflammation and improvement of alveolar capillary barrier integrity, without influencing cardiovascular haemodynamics. Cell experiments suggest that the protective effect of α_1_-AR on lung injury might arise from its suppression of inflammation through alveolar macrophages by a mechanism likely related to activation of NF-κB and ERK.

According to clinical practice, ARDS is a complication of a variety of diseases including intrapulmonary factors (e.g., infectious pneumonia and aspiration of gastric contents) and extrapulmonary factors (e.g., sepsis and haemorrhagic shock) (27). Intratracheal instillation of LPS, a method that mimics infectious pneumonia, was used to induce ARDS in mice in this study (38). Infectious pneumonia is the most prevalent cause of ARDS, accounting for 59.4% of cases (39). Moreover, studies have demonstrated that the pathological injury and functional indexes induced by this method are more consistent with experimental requirements compared with those induced by other models (40). Our results show that significant pathological damage, alveolar-capillary barrier destruction, and inflammatory response occurred in the lung tissue of ARDS mice, consistent with criteria established by the American Thoracic Society (27).

At present, limited information is available about the effect of α_1_-AR on lung injury induced by infectious stimuli such as LPS. Our results show that lung pathological damage and albumin levels in BALF were gradually mitigated with increased concentrations of PE. Moreover, this effect of PE could be partially reversed by further treatment with PRA, an α_1_-AR blocker. Previously, Satoshi Fukuda et al. explored the effect of PE on burn and smoke inhalation-induced acute lung injury of goats (24). Although there are differences in these animal models, their results showed that aerosol inhalation of PE could reduce pulmonary vascular permeability. Similarly, there was no significant difference in W/D ratio in their study either. Nai-Jing Li et al. found that intrapulmonary instillation of PE increased alveolar fluid clearance in ventilator-induced lung injury rats, and PRA abolished this effect too (23). Collectively, these studies and our findings indicate that α_1_-AR stimulation exerts an important protective effect on lung injury induced by various factors, and this protective effect is mainly related to barrier permeability and fluid balance in the lungs.

Before exploring protective mechanisms of α_1_-AR, the physiological function of α1-AR should first be considered. α_1_-AR is widely known as an important sympathetic neurotransmitter receptor capable of constricting blood vessels (41). PE is usually used to treat septic shock (42), episodes of paroxysmal supraventricular tachycardia, and hypotension during general anaesthesia and spinal anaesthesia (29). Notably, sympathetic overstimulation can lead to neurogenic pulmonary oedema – a disease similar to ARDS (43). Therefore, PE and PRA were delivered by intratracheal injection to avoid affecting the circulatory system as much as possible, and cardiovascular haemodynamics were monitored in this study. Our results show that LPS (2 mg/kg) disrupted haemodynamic stability, consistent with clinical practice, while neither PE nor PRA affected HR, SP, DP, or MAP of ARDS mice. These results suggest that LPS, a powerful toxin produced by bacteria, caused circulatory system dysfunction. Furthermore, intratracheal injection of PE or PRA appeared to mainly affect lung tissues without entering the bloodstream, thereby minimizing the impact on heart and blood vessels. Similarly, it was reported that PE intratracheal infusion had no effect on haemodynamics in goats with burn and smoke inhalation-induced lung injury at multiple time points (from 3 to 48 hours) (24). Thus, the beneficial effect of PE was unlikely associated with its effects on haemodynamics.

Damage of alveolar-capillary barriers caused by uncontrolled inflammation is considered a central mechanism of ARDS (44). Many studies aimed to reduce lung injury by inhibiting excessive inflammatory responses. Unfortunately, few reported effects of α_1_-AR on lung inflammation; indeed, only Satoshi Fukuda’s results show that nebulized PE tended to decrease IL-8 concentrations in BALF (24). We further found that α_1_-AR stimulation reduced numbers of inflammatory cells and levels of TNF-α, IL-6, and CXCL2/MIP-2 in BALF of ARDS mice. It should be noted that there were far more neutrophils than macrophages in the BALF of ARDS mice, although both alveolar macrophages and neutrophils are crucial inflammatory cells related to its occurrence. Under normal conditions (as shown for the control group in in **Figure 2A**), only a small number of macrophages (average seven per alveolar) were observed (45). Following LPS stimulation, there was an inflammatory response in the lung that caused recruitment of neutrophils, mainly under the induction of chemokines and cytokines secreted by macrophages. Because increased neutrophil numbers are considered one of the most relevant features of ARDS (27), total cells and neutrophils in BALF were detected in this study. In addition, previous studies suggested that PE treatment could decrease TNF-α and IL-6 levels in the plasma and alleviate myocarditis of sepsis rats (46, 47). Combined with the results described above, it seems likely that the lung protective effect of α_1_-AR derives from its ability to promote proper immune homeostasis by suppressing inflammatory responses.

Although macrophages, neutrophils, alveolar epithelium, pulmonary microvascular endothelium, and other cells participate in the formation of excessive inflammation during the development of ARDS (48), alveolar macrophages play a predominant role (49). Alveolar macrophages are intrinsic resident cells in alveoli and their depletion has been shown to improve IgG immune complex-induced lung injury by attenuating inflammation (50). MH-S, a continuous alveolar macrphage cell line from mice established by Mbawuike and Herscowitz in 1989 (51), is widely used in studies of bacterial pneumonia (52), chronic obstructive pulmonary disease (53), asthma (54) and ARDS (55) because it retains complete functional characteristics from parent alveolar macrophage. Thus, the alveolar macrophage cell line MH-S was used to detect the anti-inflammatory effect of α_1_-AR *in vitro*. Our results show that α_1_-AR stimulation suppressed the release of TNF-α, IL-6, and CXCL2/MIP-2, and promoted the release of IL-10 from LPS-stimulated alveolar macrophages. To determine whether this phenomenon was related to the effect of α_1_-AR stimulation on cell viability, we measured cytotoxicity with a CCK-8 kit and live/dead staining. The results show that cell viability was not affected. Previously, Laurel A. Grisanti et al. found that PE could significantly reduce expression of TNF-α, IL-8, and MIP-1β in human THP-1 monocytes stimulated with LPS, consistent with our research (25). However, their results also showed that PE could promote expression of IL-1β, an important proinflammatory cytokine with wide biological effects (26). We wondered if the same change in expression of IL-1β occurred in our study, but found that LPS did not induce IL-1β release from MH-S cells (data not shown). In addition, Hongmei Li et al. used PE and PRA to verify that α_1_-AR stimulation could inhibit TNF-α secretion from cardiomyocytes in LPS-stimulated rats (56), which further confirmed the reliability of our findings. Interestingly, a previous study showed that PE dose-dependently attenuated TNF-α production and enhanced IL-10 release in isolated primary human monocytes (57). In contrast to the current findings, β-AR rather than α_1_-AR was thought to mediate the potent anti-inflammatory effects of PE. However, what must be emphasized is that PE is considered as a specific α_1_-AR agonist in clincal and basic research (58). Thus, we chose PE to study the effects of α_1_-AR on ARDS. Moreover, PRA (a apecific of α_1_-AR antagonist) reversed the effect of PE in our study, further confirming a role for α_1_-AR in this process. Importantly, its potential to activate other receptors (α_2_ or β), is worth exploring.

The occurrence of uncontrolled lung inflammation involves a complex signalling pathway network connected by important nodes and hubs. NF-κB is a known core molecule, while MAPKs and Akt are focal points (59). All of these signalling pathways may be involved in the regulation of inflammatory responses by α_1_-AR (26, 46, 47, 56). Accordingly, to clarify the specific mechanism by which the anti-inflammatory effect of α_1_-AR occurs in alveolar macrophages, we detected all the molecules described in the studies above under the condition of different concentrations of PE. Finally, according to our results, we concluded that α_1_-AR stimulation inhibited NF-κB activation and enhanced ERK1/2 activation without significantly influencing p38, JNK, or Akt activation in LPS-stimulated MH-S cells. Research by Laurel A. Grisanti showed that PE increases IL-1β expression in LPS-stimulated human monocytes and macrophages by promoting activation of p38 rather than ERK1/2 or JNK (26). Other reports suggest that α_1_-AR stimulation suppressed inflammation by inhibiting p38 and NF-κB activation, but enhanced activation of ERK1/2 in LPS-stimulated cardiomyocytes (46) and Akt in myocardium from septic animals (47). According to these studies, aspects of inflammatory regulation and molecular mechanisms related to α_1_-AR may completely differ across various cells, tissues, and disease conditions. In addition, we also noticed that there was a higher dose of PE was used in the research of Laurel A. Grisanti than that of other researches which might lead to different results. Overall, the reported activation of ERK1/2 and NF-κB in previous studies was consistent with our results; in particular, NF-κB is a prototypical proinflammatory transcription factor that plays important roles in the expression of numerous cytokines and chemokines, and many studies have demonstrated its involvement in the occurrence and development of ARDS (60, 61). The observed activation of NF-κB in this study is consistent with changes of cytokines, suggesting that α_1_-AR activation may alleviate lung injury by inhibiting NF-κB activation. The ERK1/2 pathway was previously reported to participate in marcophage activation, although its effect in this process was debatable (62, 63). What is clear is that stimulation of α_1_-AR could alleviate sepsis-induced cardiomyocyte apoptosis and inflammation by activating ERK1/2 pathway (46, 56). Moreover, evidence suggests that ERK1/2 activation could participate in M2-like macrophages polarization (64). Collectively, these studies identify a relationship between α_1_-AR and ERK1/2 pathway. Our current study has provided preliminary information showing that NF-κB and ERK1/2 in the protective effect of α_1_-AR on lung injury.

Here we conducted a comprehensive and systematic study to evaluate the role of α_1_-AR from several aspects, including pathological damage, barrier disruption, inflammatory responses, and molecular mechanism in ARDS. The results further expanded our understanding of the effect of sympathetic nervous system in ARDS and provided basic research clues for its clinical treatment. Certainly, it is undeniable that there are still many limitations of this study that need to be solved in the future. First, haemodynamics were only monitored before establishing ARDS model and sacrificing of mice, and did not show evident changes after treatment with PE or PRA. If continuous and effective monitoring was carried out, these results would be more convincing. Second, the observed changes of cytokines imply that there might be a relationship between α_1_-AR and macrophage polarization. Because TNF-α, IL-6, CXCL2/MIP-2, and IL-10 are phenotypical markers of M1 or M2 macrophages, our results suggest that α_1_-AR stimulation could potentially transform alveolar macrophages from an M1 to M2 subtype. To verify this conjecture, we also detected other typical markers of macrophage polarization, including CD86 and iNOS for the M1 subtype, and CD206 and Arg-1 for the M2 subtype. Confusingly, none of the molecules were detected in MH-S cells after stimulation with LPS (data not shown). We considered that this might be related to our experimental conditions, such as the LPS concentration or time-period of stimulation. In addition, detection of number and type of macrophages in BALF may also provide a more accurate reflection of the role of macrophages in the protective effect of PE against LPS. However, the number of macrophages in BALF was too few to be detected in some samples. Finally, and more importantly, the molecular mechanisms underlying the effects of α_1_-AR in lung injury need to be further explored. At present, no other research has verified the involvement of NF-κB, Akt, and MAPK signalling pathways in alveolar macrophages with activated α_1_-AR. Although we confirmed that NF-κB and ERK1/2 participated in the mechanism of the protective effect of α_1_-AR, many other factors could also be involved.

In summary, our study demonstrated that α_1_-AR stimulation ameliorates LPS-induced lung injury by suppressing excessive inflammatory responses in lung tissues without affecting haemodynamics. The mechanism of anti-inflammation elicited by α_1_-AR stimulation may be associated with its ability to inhibit alveolar macrophage activation by suppressing NF-κB activation and promoting ERK1/2 activation.

## Data availability statement

The original contributions presented in the study are included in the article/supplementary material. Further inquiries can be directed to the corresponding authors.

## Ethics statement

The animal study was reviewed and approved by Animal Care and Scientific Committee of Peking University Health Science Centre (Approval No: SA2020336).

## Author contributions

ZC designed and performed the experiments, collected and analyzed data, and drafted and approved the final version. CY, ZZ, FZ, and ZS performed experiments and data analysis. CW designed the experiments and revised the article. XZ and HX supervised the research design, revised the article, and approved the final version. All authors contributed to the article and approved the submitted version.
